# GABA Prevents Age-Related Sarcopenic Obesity in Mice with High-Fat-Diet-Induced Obesity

**DOI:** 10.3390/cells12172146

**Published:** 2023-08-25

**Authors:** Heegu Jin, Hyun-Ji Oh, Boo-Yong Lee

**Affiliations:** Department of Food Science and Biotechnology, College of Life Science, CHA University, Seongnam 13488, Republic of Korea; heegu94@hanmail.net (H.J.); guswl264@naver.com (H.-J.O.)

**Keywords:** sarcopenic obesity, obesity, sarcopenia, energy expenditure, insulin resistance, muscle protein degradation, GABA

## Abstract

Sarcopenic obesity is characterized by concurrent obesity and muscle wasting (sarcopenia) and is common in the elderly. Sarcopenic obesity has steadily increased as the aging population has grown and is an increasing public health burden. Both obesity and sarcopenia independently increase health risks of the elderly, but sarcopenic obesity has a greater effect on metabolic disease than either obesity or sarcopenia alone. The metabolic mechanisms of obesity and sarcopenia are strongly interconnected, and obesity and sarcopenia form a vicious cycle, with each pathology exacerbating the other. The pathogenesis of sarcopenic obesity is more complex than either disease alone and remains incompletely understood, underscoring the significant unmet clinical need for effective sarcopenic obesity treatments. We aimed to determine the efficacy and underlying regulatory mechanisms of Gamma-aminobutyric acid (GABA) in sarcopenic obesity in high-fat-diet-fed obese aged mice and alterations in related mechanisms to determine the potential of GABA as a therapeutic modality for sarcopenic obesity. In this study, we used young (3 months) and aged (20 months) mice to evaluate age-related sarcopenic obesity. The daily administration of GABA for 8 weeks resulted in decreased fat mass and increased muscle mass and strength in aged mice. GABA also enhanced energy expenditure in both adipose tissue and skeletal muscle. In addition, GABA promoted muscle synthesis and decreased muscle degradation by activating the phosphatidylinositol-3-kinase (PI3K)/Akt pathway. These findings demonstrate that GABA has potential uses in preventing age-related sarcopenic obesity and related metabolic diseases.

## 1. Introduction

Obesity is a global public health concern that increases the early onset of numerous metabolic diseases [1]. The prevalence of obesity in the elderly has steadily increased, as the aging population grows, making sarcopenic obesity a growing public health burden [2]. During the aging process, two major changes in body composition occur, with the progressive loss of skeletal muscle mass (sarcopenia) and a progressive increase in body fat (obesity) [3]. The co-occurrence of these two changes, sarcopenic obesity, is related to physical inactivity and increases the risk of metabolic disease more significantly than obesity or sarcopenia alone [4]. Therefore, addressing sarcopenic obesity is important for preventing metabolic pathology and physical disability in elderly persons at a high risk of metabolic disease [5].

Sarcopenic obesity is the combination of obesity and sarcopenia, in which skeletal muscle mass and strength decline in parallel with the increased body fat in the elderly [6]. In the pathology of age-related obesity, fat distribution changes, with increased visceral fat and decreased subcutaneous fat [7]. The excessive accumulation of visceral fat with aging increases the risk of triggering and aggravating the pathogenesis of sarcopenic obesity [8]. This could occur because visceral fat contains more large adipocytes with higher lipid metabolism rates, which increases circulating free fatty acids, promoting dyslipidemia and metabolic syndrome [9,10]. 

Increased visceral fat mass is a primary cause of sarcopenia, as skeletal muscle is a major metabolic site for the regulation of energy homeostasis and is important for the utilization of lipids and glucose as fuel substrates [11]. Obesity- and age-related decreases in the components of energy expenditure are associated with impaired lipid and glucose homeostasis, which are primarily caused by mitochondrial dysfunction and the decreased oxidative capacity of skeletal muscle mitochondria [12]. Uncoupling protein 3 (UCP3) regulates energy expenditure and mitochondrial function, and it is localized in the inner membrane of skeletal muscle mitochondria and increases fatty acid β-oxidation [13,14].

Imbalanced energy consumption is linked to lipid and glucose metabolic disorders in both adipose tissue and skeletal muscle, especially in the context of aging [15]. The synergistic effects of obesity and sarcopenia on glucose metabolism and insulin sensitivity cause dyslipidemia, glucose intolerance, and insulin resistance [16]. As obesity progresses, adipocyte hyperplasia and hypertrophy increase, such that excessive lipid accumulation in adipose tissue increases serum lipid levels and flux in the skeletal muscle, promoting insulin resistance and muscle atrophy [17]. Insulin is the central regulator of glucose metabolism in both adipose tissue and skeletal muscle, and insulin binding to its receptor initiates intracellular insulin signaling [18]. This signaling is initiated by insulin and is primarily regulated by the phosphatidylinositol-3-kinase (PI3K)/Akt (protein kinase B) pathway [19]. PI3K phosphorylates Akt, and activated Akt subsequently stimulates the membrane translocation of glucose transporter 4 (GLUT4) to facilitate glucose uptake [20].

Obesity also disrupts muscle protein turnover via the PI3K/Akt pathway, which impairs the balance between muscle protein synthesis and degradation in skeletal muscle and causes a progressive decline in skeletal muscle mass and function [21]. As a major underlying mechanism of sarcopenic obesity, the PI3K/Akt pathway is a central link between insulin metabolism and skeletal muscle atrophy [22]. When this pathway is suppressed, the development and progression of sarcopenia become worse, in addition to affecting the balance between muscle protein synthesis and degradation [23]. Activation of the PI3K/Akt pathway promotes muscle synthesis and decreases muscle degradation [24]. Akt inhibits muscle protein degradation, as do decreased levels of myostatin, a negative regulator of muscle mass that accelerates muscular atrophy [25]. A series of pathways, such as the phosphorylation of forkhead box O3a (FoxO3a), downregulates transcription factors of muscle-specific E3 ubiquitin ligases, including atrogin-1 and muscle ring finger 1 (MuRF1) [26]. 

Fat accumulation and aging dysregulate the production of adipokines and pro-inflammatory cytokines, resulting in chronic low-grade inflammation [27]. The inflammatory factors, including TNF-α, IL-1β, and IL-6, are primarily produced by visceral fat and exacerbate the development and progression of muscle dysfunction and insulin resistance, worsening sarcopenic obesity [28]. 

Gamma-aminobutyric acid (GABA) is a widely distributed four-carbon free amino acid that is abundant in vegetables, fruits, and fermented foods [29]. GABA is produced from l-glutamic acid by glutamate decarboxylase and functions as a major inhibitory neurotransmitter in the central nervous system [30]. The role of GABA in the central nervous system is associated with the regulation of synaptic transmission, improving sleeplessness and depression, enhancing immunity, and regulating insulin secretion [31]. Recent studies have shown GABA to be beneficial, but the effects of GABA on age-related sarcopenic obesity in HFD-fed aged mice have not been studied in detail. Notably, multiple biological activities of GABA were reported due to insulin metabolism, and GABA-receptor-mediated signaling is linked to body weight and skeletal muscle growth and maintenance [32]. This suggests that GABA is a potential therapeutic for the prevention and treatment of sarcopenic obesity. However, the therapeutic effects of GABA on obesity associated with age-related sarcopenia, as well as the underlying regulatory mechanisms, remain unclear. Therefore, in the present study, we aimed to evaluate whether GABA could attenuate sarcopenic obesity using aged mice with high-fat diet (HFD)-induced obesity. These findings suggest that GABA can prevent age-related sarcopenic obesity and its related metabolic syndrome by reducing fat mass and increasing muscle mass.

## 2. Materials and Methods

### 2.1. Preparation of GABA

GABA (A5835; Sigma-Aldrich, St. Louis, MO, USA) was dissolved in distilled water, and we administered GABA via oral gavage to each of the mice (10 or 30 mg/kg/day). 

### 2.2. Experimental Animals and Treatments

Animal experiments were performed according to the criteria outlined in the “Guide for the Care and Use of Laboratory Animals” of the National Academy of Science, published by the National Institutes of Health, and were approved by the Institutional Animal Care and Use Committee of CHA University (Approval Number 220145). Male C57BL/6J mice aged 3 months or 20 months were purchased from Raon Bio (Yongin, Republic of Korea) and housed at 3 mice per cage in a temperature- and humidity-regulated facility with a 12 h light/dark cycle. After 1 week of adaptation, mice were randomized into six groups (*n* = 9 per group): young chow diet (CD)-fed mice (YM CD); young HFD-fed mice (YM HFD); aged CD-fed mice (AM CD); aged HFD-fed mice (AM HFD); aged HFD-fed that received 10 or 30 mg/kg/day GABA (AM GABA10 and AM GABA30, respectively) for 8 weeks. During the study period, body mass, dietary intake, fasting blood glucose, rectal temperature, and grip strength were measured weekly at the same time of the day. At the end of the experiment, mice were fasted for 12 h and euthanized with CO_2_, and tissue samples collected, immediately weighed, and stored at −80 °C until further analysis.

### 2.3. Body Mass Measurement

The body masses of the mice were measured weekly using an analytical balance.

### 2.4. Rectal Temperature Measurement

Mouse rectal temperature was measured weekly at 10:00 am using a Testo 925 Type Thermometer (Testo, Lenzkirch, Germany).

### 2.5. Fasting Blood Glucose Measurement

The fasting glucose concentrations in blood samples (0.6 μL) collected weekly from the tail vein using tail vein puncture after 12 h of fasting were measured using an Accu-Check Blood Glucose Meter (Roche, Basel, Switzerland).

### 2.6. Oral Glucose Tolerance Testing

Oral glucose tolerance testing was performed after an overnight fast. Mice received 1.5 g/kg of D-glucose orally, and blood glucose was measured after 0, 30, 60, 90, and 120 min using an Accu-Check Blood Glucose Meter.

### 2.7. Grip Strength Measurement

Mouse grip strengths were measured weekly using a Chatillon Force Measurement System (Columbus Instruments, Columbus, OH, USA).

### 2.8. Dual-Energy X-ray Absorptiometry

Mouse body composition, including fat and lean mass, was analyzed using dual-energy X-ray absorptiometry (DEXA) with an InAlyzer dual X-ray absorptiometer (Medikors, Seongnam, Republic of Korea). The visceral fat and hindlimb, including the quadriceps (QUA) and gastrocnemius muscle (GAS), were selected as the regions of interest (ROI).

### 2.9. Biochemical Analysis

After 8 weeks of GABA treatment, blood samples were obtained through cardiac puncture and centrifuged at 3000× *g* for 20 min at 4 °C, and serum samples were stored at −80 °C until subsequent analysis. Serum insulin concentrations were measured using a Mouse Metabolic Hormone Magnetic Bead Panel (Merck Millipore, Burlington, MA, USA). Serum concentrations of TG, total cholesterol, low-density lipoprotein (LDL)-cholesterol, and high-density lipoprotein (HDL)-cholesterol were determined using colorimetric assay kits (Roche). Serum concentrations of TNF-α and IL-1β were measured using a Mouse High-sensitivity T Cell Magnetic Bead Panel (Merck Millipore, Burlington, MA, USA). Serum testosterone concentrations were measured using a Mouse Testosterone ELISA Kit (Abcam, Cambridge, UK).

### 2.10. Histological Analysis

Samples of visceral adipose tissue (VAT), QUA, and GAS were fixed in 4% paraformaldehyde and embedded in paraffin. Sections were stained with hematoxylin and eosin (H&E) and examined using a Nikon E600 microscope (Nikon, Tokyo, Japan).

### 2.11. Immunofluorescence

Samples of VAT, QUA, and GAS were deparaffinized and incubated with anti-PKA, anti-UCP1, anti-MyoD, or anti-UCP3 antibodies. Secondary anti-mouse fluorescein isothiocyanate (FITC)-conjugated and anti-rabbit Alexa Fluor™ 594-conjugated antibodies were then applied. DAPI (Thermo Fisher Scientific, Waltham, MA, USA) was used to stain the cell nuclei. Fluorescent images were captured using a Zeiss confocal laser scanning microscope (LSM880; Carl Zeiss, Oberkochen, Germany) with Zen 2012 software (Version 1.1.1.0, Carl Zeiss).

### 2.12. Western Blot Analysis

Samples of VAT, QUA, and GAS were lysed in lysis buffer (iNtRON Biotechnology, Seoul, Republic of Korea) containing phosphatase and protease inhibitors. Lysate protein concentrations were quantified using a protein assay kit (Bio-Rad, Hercules, CA, USA). Lysates containing equal amounts of protein (20 μg) were separated using 8–12% SDS-PAGE, and proteins were electrotransferred to polyvinylidene fluoride membranes. Membranes were then incubated with primary antibodies (1:1000 dilution) overnight at 4 °C and exposed to horseradish peroxidase-conjugated secondary antibodies (1:5000 dilution). Antibodies targeting C/EBPα (sc61), PPARγ (sc7273), FABP4 (sc30088), sterol regulatory element-binding protein 1 (SREBP1, sc366), LPAATθ (sc68372), lipin 1 (sc98450), DGAT1 (sc32861), phosphorylated PKA (p-PKA, Ser 114, sc136460), PGC1α (sc13067), phosphoinositide 3-kinase (PI3K, sc7174), MyoD (sc760), myogenin (sc12732), myocyte enhancer factor 2 (MEF-2, sc313), and glyceraldehyde 3-phosphate dehydrogenase (GAPDH, sc365062) were purchased from Santa Cruz Biotechnology (Dallas, TX, USA). Antibodies targeting ATGL (cs2138), phosphorylated HSL (p-HSL, Ser 563, cs4139), phospho-AKT (p-AKT, Ser 473, cs9271), AKT (cs9272), phospho-forkhead box O3 (p-FoxO3a, Ser 253, cs13129), FoxO3a (cs2497), phospho-mTOR (p-mTOR, Ser 2448, cs2971), p-4EBP1 (Thr37/46, cs2855), TNF-α (cs3707), and IL-1β (cs12426) were purchased from Cell Signaling Technology (Danvers, MA, USA). Antibodies targeting MGL, PPARα (ab24509), PRDM16 (ab202344), UCP1, NRF1 (ab175932), TFAM (ab131607), UCP3 (ab10985), p-PI3K (Tyr 607, ab182651), GLUT4 (ab654), Atrogin-1 (ab168372), MuRF1 (ab172479), myostatin (ab203076), and Myf5 (ab125301) were purchased from Abcam, and an antibody targeting Myf6 (PA5-51016) was purchased from Invitrogen (Carlsbad, CA, USA).

### 2.13. Statistical Analysis

Data are expressed as the mean ± SEM (*n* = 8), and comparisons were made using one-way ANOVA followed by Tukey’s post-hoc comparison (IBM SPSS Statistics Version 20.0, Armonk, NY, USA). *p* < 0.05 was considered statistically significant.

## 3. Results

### 3.1. Effect of HFD-Induced Obesity and Aging on the Body Composition of Young and Aged Mice

**Figure 1:**
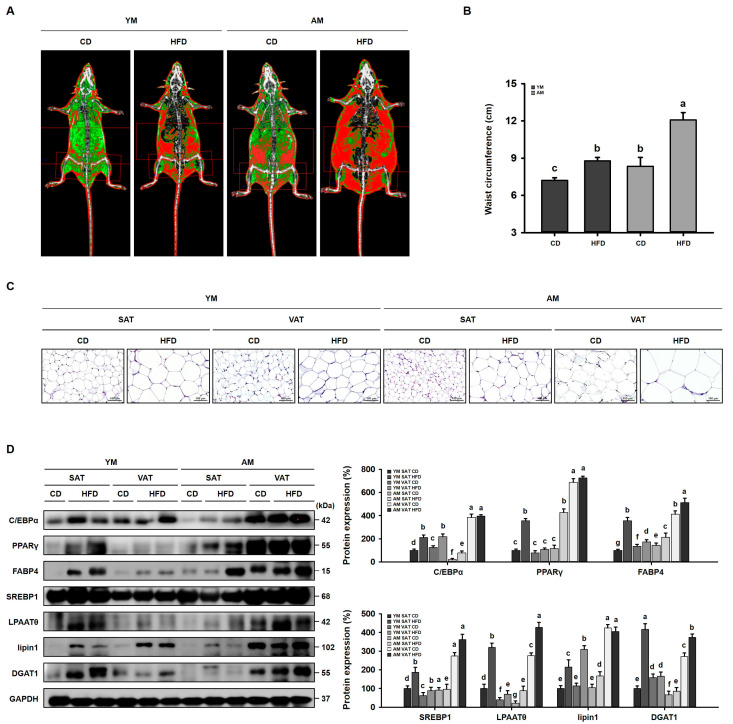
Effects of obesity and age-related sarcopenic obesity in aged mice. (**A**) Body composition images obtained using DEXA. Fat tissue is shown in red and lean is in green. (**B**) Waist circumference after 8 weeks of HFD treatment. (**C**) H&E staining of SAT and VAT. Scale bar: 100 μm. (**D**) Western blots of adipogenic proteins (C/EBPα, PPARγ, and FABP4) and lipogenic proteins (SREBP1, LPAATθ, lipin1, and DGAT1) in the SAT and VAT. Values with different letters are significantly different: *p* < 0.05 (a > b > c > d > e > f > g).

To evaluate the effects of GABA on obesity- and age-related sarcopenic obesity, we fed an HFD for 8 weeks to both young (3 months) and aged (20 months) mice to determine differences in the body composition, as the effects of obesity change with age. The masses of subcutaneous and VAT significantly differed between young and aged mice (Figure 1A). A characteristic age-related change in fat distribution is that the amount of visceral fat increases, while the amount of subcutaneous fat decreases. Moreover, for waist circumference shown in Figure 1B, after 8 weeks of HFD, the waist circumference of both young and aged mice was higher than that of mice fed the CD. However, visceral obesity was more severe in aged HFD-fed mice than in young HFD-fed mice. Furthermore, histological analysis showed that adipocyte size was increased in the VAT compared to that in the SAT of HFD-fed aged mice (Figure 1C). We next performed Western blot analysis to investigate the molecular mechanisms of fat accumulation in this context, which is regulated by adipogenesis and lipogenesis. In aged HFD-fed mice, adipogenic transcription factors and lipogenic factors were higher in VAT than in SAT, suggesting that fat accumulation was increased in the VAT of HFD-fed aged mice (Figure 1D).

Interestingly, we observed similar DEXA patterns of lean hindlimb mass between young HFD-fed mice and aged CD-fed mice, revealing that both obesity and aging decreased skeletal muscle mass (Figure 1A). This suggests that HFD-induced obesity could also cause sarcopenia in young mice. However, the increase in HFD-induced visceral obesity with aging more severely impacted the loss of muscle mass. Therefore, we used a mouse model of sarcopenic obesity in which aged mice were fed with an HFD, inducing both obesity and sarcopenia, and then further analyzed the VAT, QUA, and GAS to evaluate the link between the two primary tissue types affected by HFD- and age-associated changes in body composition.

### 3.2. GABA Prevents HFD-Induced Visceral Obesity in Aged Mice

**Figure 2:**
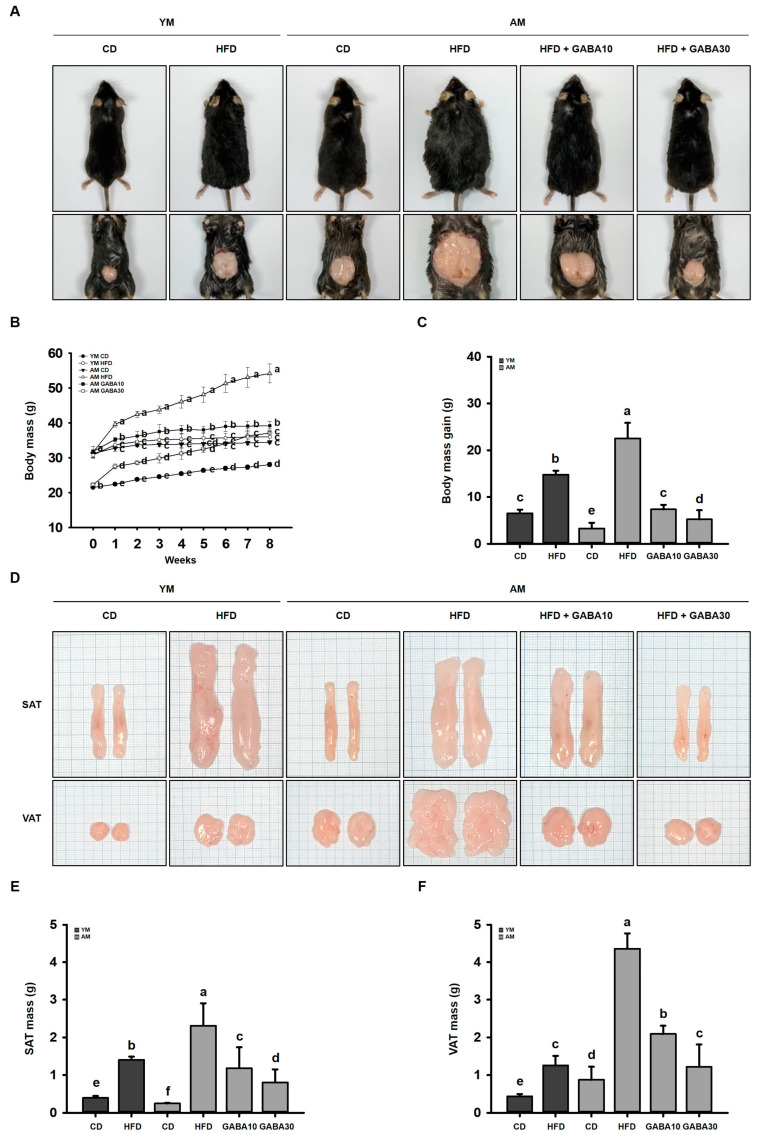
Effects of GABA on obesity in aged HFD-fed mice. (**A**) Representative images of mice. (**B**) Body mass during 8 weeks of GABA treatment. (**C**) Body mass gain after 8 weeks of GABA treatment. (**D**) Representative images of SAT and WAT. The scale of one grid is 1 mm. (**E**) SAT mass and (**F**) VAT mass after 8 weeks of GABA treatment. Values with different letters are significantly different: *p* < 0.05 (a > b > c > d > e > f).

To investigate if GABA affected visceral obesity in aged mice, we fed aged mice a CD or HFD. HFD-fed mice received oral vehicle control or GABA (10 or 30 mg/kg/day) for 8 weeks. At the experimental endpoint, representative images of mice suggested that HFD-fed mice were visibly larger than both young and aged CD-fed mice (Figure 2A). Body mass gain was more severe in aged mice, but GABA treatment dose-dependently decreased the body mass gain and especially protected against visceral obesity (Figure 2A–C). Consistent with this result, GABA treatment reduced VAT accumulation in aged mice (Figure 2D). Importantly, in young mice, fat accumulation was higher in the SAT than in the VAT, which was also decreased by GABA (Figure 2E,F).

### 3.3. GABA Decreased Adipocyte Size and Inhibits Lipid Accumulation in Aged Mice

**Figure 3:**
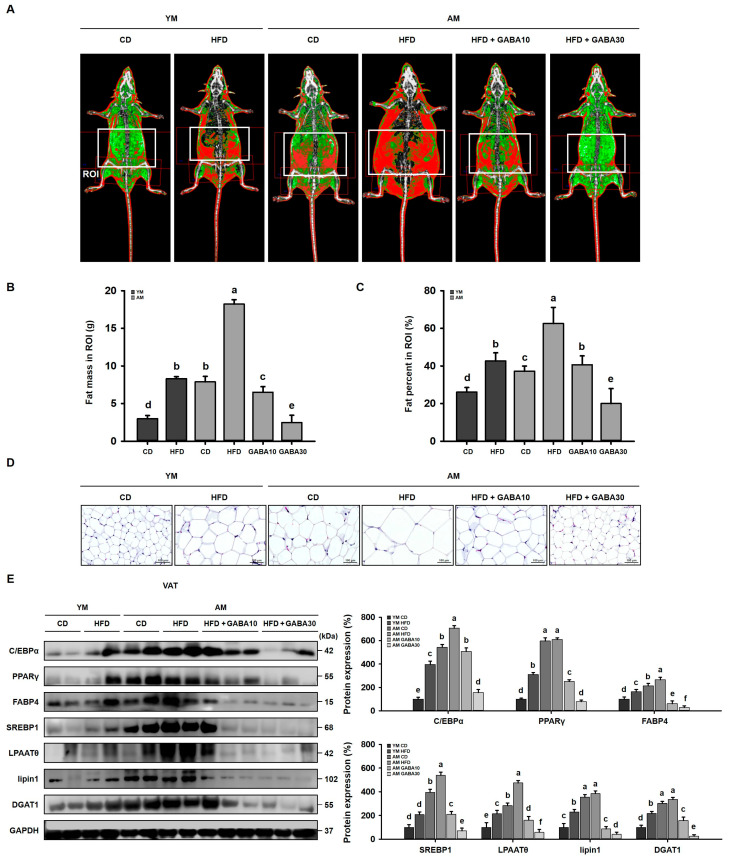
Effects of GABA on visceral lipid accumulation in aged HFD-fed mice. (**A**) Representative DEXA images of visceral fat. (**B**) Fat mass and (**C**) fat percentage in ROIs. (**D**) H&E staining of VAT. Scale bar: 100 μm. (**E**) Western blots of adipogenic proteins (C/EBPα, PPARγ, and FABP4) and lipogenic proteins (SREBP1, LPAATθ, lipin1, and DGAT1) in the VAT. Values with different letters are significantly different: *p* < 0.05 (a > b > c > d > e > f).

To determine the effect of GABA on VAT mass, fat mass was measured using DEXA (Figure 3A). The fat mass and percentage in the ROIs of the HFD groups were higher than those of the CD groups, and GABA treatment dose-dependently lowered the fat mass (Figure 3B,C). Moreover, H&E staining demonstrated that GABA dose-dependently decreased the adipocyte size of the VAT in HFD-fed aged mice (Figure 3D). To assess adipogenesis and lipogenesis in the VAT of aged mice, we measured the protein expression via Western blot analysis. Protein levels of adipogenesis and lipogenesis factors in the VAT were significantly higher in aged HFD-fed mice than in young HFD-fed mice (Figure 3E). GABA dose-dependently decreased the expression of adipogenic and lipogenic proteins. Taken together, these findings suggested that GABA inhibits lipid accumulation by decreasing VAT adipogenesis and lipogenesis.

### 3.4. GABA Increased Body Temperature and Energy Expenditure in Aged Mice

**Figure 4:**
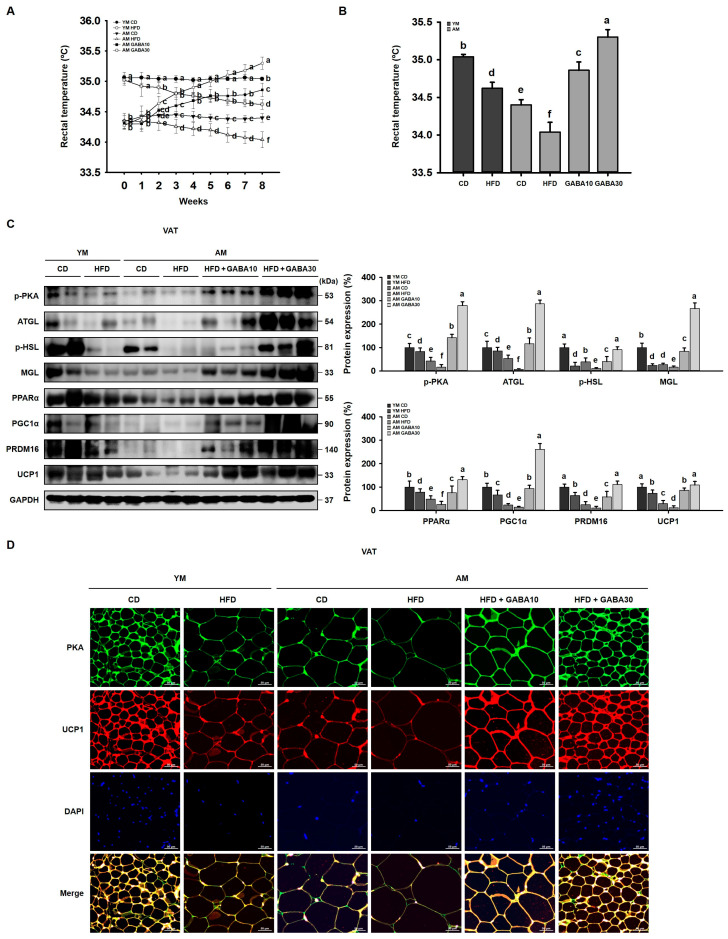
Effects of GABA on lipolysis and browning in the VAT of aged HFD-fed mice. (**A**) Rectal temperature during the 8-week study period. (**B**) Rectal temperature after 8 weeks of GABA treatment. (**C**) Western blots of lipolytic enzymes (p-PKA, ATGL, p-HSL, and MGL) and browning proteins (PPAR α, PGC1α, PTDM16, and UCP1) in the VAT. (**D**) Immunofluorescence images of the expression of PKA (green) and UCP1 (red) in the VAT. Scale bar: 50 μm. Values with different letters are significantly different: *p* < 0.05 (a > b > c > d > e > f).

To investigate the effect of GABA on energy expenditure, the mouse body temperature was measured during the 8-week study period. At the beginning of the study, body temperature was lower in aged mice than in young mice, and during the experimental period, the HFD group lost their thermogenic ability, as represented by the decreased rectal temperature (Figure 4A,B). However, rectal temperatures were higher in GABA-treated mice, indicating that GABA increased energy expenditure via heat generation. Therefore, to determine if GABA stimulated lipolysis and thermogenic gene expression and promoted adipose browning, we subsequently performed Western blot analyses. Aged HFD-fed mice exhibited lower levels of lipolytic enzymes and thermogenic proteins than did young HFD-fed mice or aged CD-fed mice (Figure 4C). However, these proteins were upregulated by GABA. In addition, the intensity of PKA and UCP1 immunofluorescence was higher in the VAT from GABA-treated aged mice (Figure 4D). Taken together, these findings suggested that GABA promoted browning via lipolysis in the VAT, which results in energy loss via heat.

### 3.5. GABA Stimulation of Skeletal Muscle Mitochondrial Biogenesis in the Skeletal Muscle of Aged Mice

**Figure 5:**
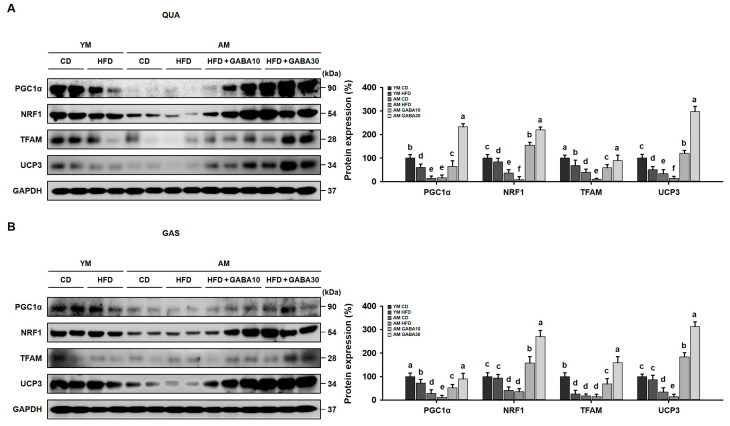
Effects of GABA on skeletal muscle mitochondrial biogenesis in the skeletal muscle of aged HFD-fed mice. (**A**) Western blots of the mitochondrial biogenesis-related proteins PGC1a, NRF1, TFAM, and UCP3 in the QUA. (**B**) Western blots of proteins involved in mitochondrial biogenesis, including PGC1a, NRF1, TFAM, and UCP3 in GAS. Values with different letters are significantly different: *p* < 0.05 (a > b > c > d > e > f).

Consistent with energy expenditure in the VAT, skeletal muscle is also energetically demanding, requiring abundant ATP to function. Mitochondria play a central role in muscle and energy metabolism [33]. Therefore, we examined the effect of GABA on skeletal muscle mitochondrial biogenesis and energy expenditure in the QUA and GAS of aged mice. In the aged HFD-fed group, protein levels of PGC1α, NRF1, TFAM, and UCP3 were significantly decreased (Figure 5A,B). However, in both the QUA and GAS, GABA significantly increased the expression of these proteins and ultimately induced the inner mitochondrial membrane transporter UCP3, which is similar to UCP1 in VAT. These findings indicated that GABA stimulated mitochondrial energy production, potentially due to enhanced UCP3 activity, increasing the oxidative capacity in aged HFD-fed mice.

### 3.6. GABA Alleviation of Dyslipidemia, Glucose Tolerance, and Insulin Resistance in Aged Mice

**Figure 6:**
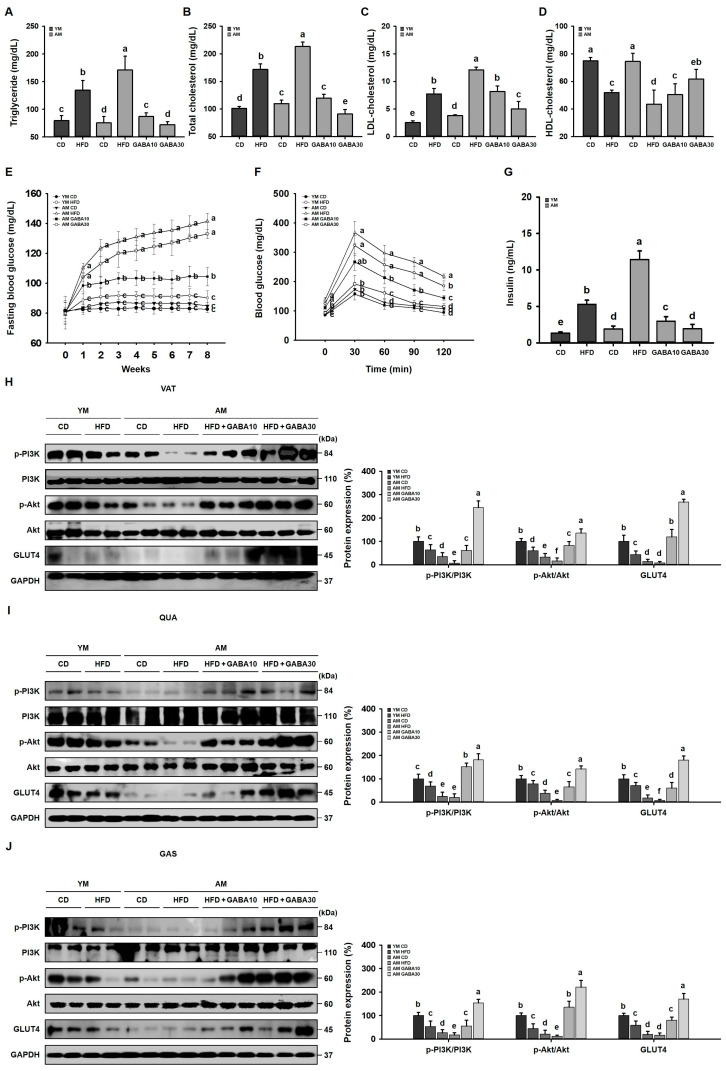
Effects of GABA on hyperlipidemia and hyperglycemia in aged HFD-fed mice. (**A**) Triglycerides, (**B**) total cholesterol, (**C**) LDL cholesterol, and (**D**) HDL cholesterol concentrations after 8 weeks of GABA treatment. (**E**) Fasting blood glucose, (**F**) oral glucose tolerance, and (**G**) serum insulin concentrations after 8 weeks. (**H**) Western blots of proteins involved in glucose uptake (p-PI3K, PI3K, p-Akt, Akt, and GLUT4) in the VAT. (**I**) Western blots of glucose uptake proteins in the QUA and (**J**) GAS. Values with different letters are significantly different: *p* < 0.05 (a > b > c > d > e > f).

HFD-induced obesity and aging are associated with hyperlipidemia and hyperglycemia in both adipose tissue and skeletal muscle, which are the main tissues that regulate lipid and glucose metabolism [34]. Therefore, we determined if GABA affected the serum lipid profile. GABA ameliorated HFD- and age-induced dyslipidemia, including the TG, total cholesterol, LDL cholesterol, and HDL cholesterol levels in aged HFD-fed mice (Figure 6A–D). Subsequently, we investigated the effect of GABA on blood glucose concentrations in aged HFD-fed mice. Over the 8-week study period, fasting blood glucose was increased gradually in the HFD groups, which was more severe in aged mice. GABA decreased fasting blood glucose, such that the concentrations in the GABA-treated HFD groups were similar to those of the CD groups (Figure 6E). GABA increased glucose tolerance in an oral glucose tolerance test (Figure 6F) and significantly decreased serum insulin levels (Figure 6G), further suggesting improved glucose tolerance and insulin sensitivity. We next performed Western blot analysis to investigate if GABA improved glucose uptake through the PI3K/Akt pathway in both the VAT and skeletal muscle. HFD feeding decreased PI3K and Akt phosphorylation in the VAT, QUA, and GAS, which were increased by GABA in aged HFD-fed mice (Figure 6H–J). In addition, GABA treatment increased the expression of GLUT4, the major insulin-sensitive glucose transporter. Taken together, these results suggest that GABA improved glucose homeostasis and insulin sensitivity in both the VAT and skeletal muscle of aged HFD-fed mice.

### 3.7. GABA Prevented the Loss of Skeletal Muscle Mass and Strength in Aged Mice

**Figure 7:**
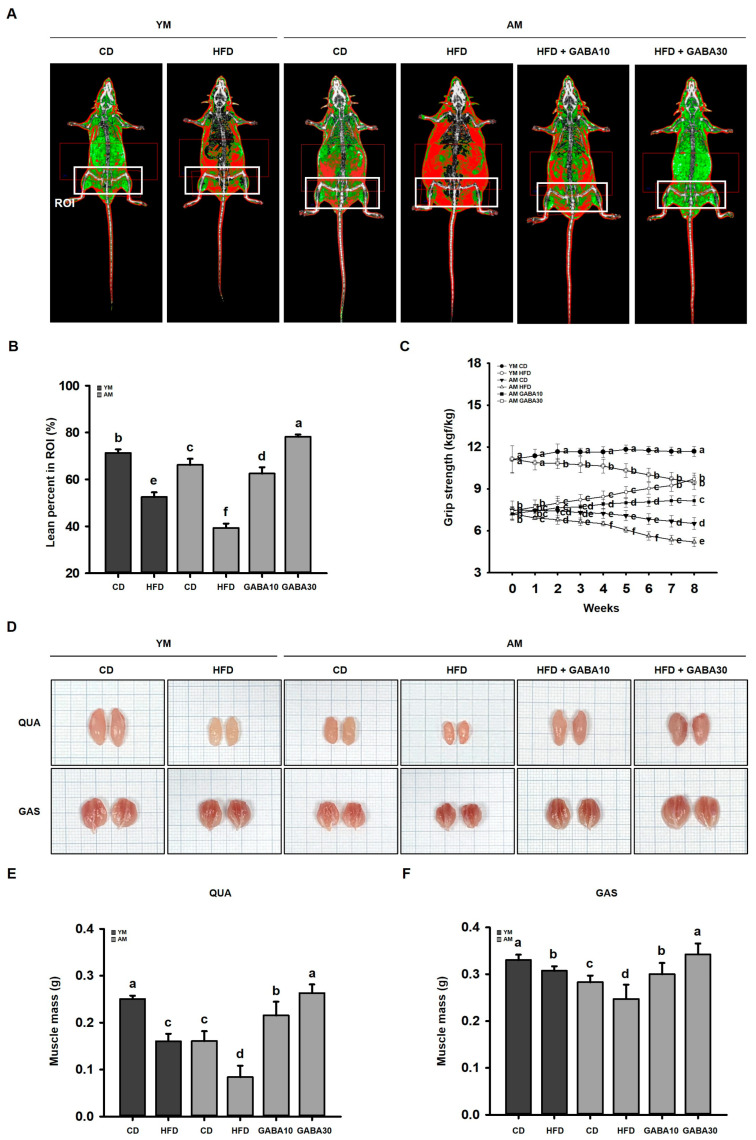
Effects of GABA on skeletal muscle mass and functions in aged HFD-fed mice. (**A**) Representative DEXA images of skeletal muscle. (**B**) Lean percentage in ROIs. (**C**) Grip strength measured weekly during the 8-week study period. (**D**) Representative images of the QUA and GAS. The scale of one grid is 1 mm. (**E**) QUA mass and (**F**) GAS mass at the end of the study period. Values with different letters are significantly different: *p* < 0.05 (a > b > c > d > e > f).

To investigate the effect of GABA on muscle mass, mouse lean mass was measured using DEXA. Because aging is associated with sarcopenia, aged CD-fed mice exhibited decreased lean mass (Figure 7A). Interestingly, the HFD decreased lean mass in both young and aged mice (Figure 7B). In aged HFD-fed mice, muscle mass was decreased compared to that in mice challenged with HFD feeding or aging alone. Lean mass was increased in the GABA-treated groups in a dose-dependent manner. Similarly, exercise capacity, determined based on grip strength, was decreased in the HFD group, but GABA steadily strengthened the grip strength in aged HFD-fed mice (Figure 7C). Although sarcopenic obesity decreased muscle sizes and masses of the QUA and GAS, it was inhibited by GABA treatment in aged HFD-fed mice (Figure 7D–F).

### 3.8. GABA Decreased Sarcopenic Muscle Protein Degradation in Aged Mice

**Figure 8:**
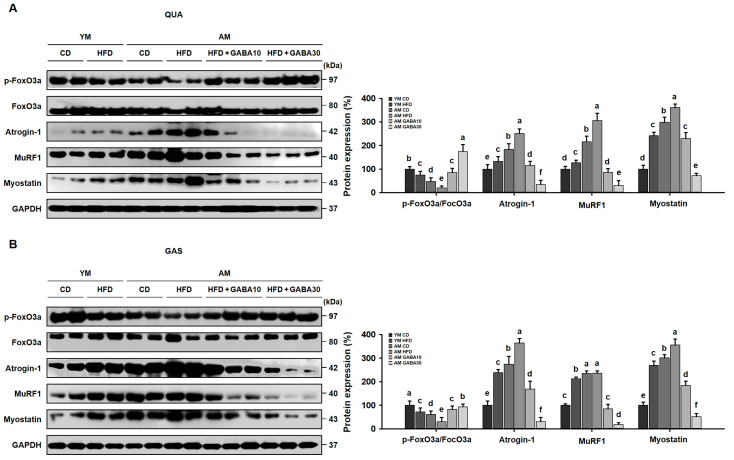
Effects of GABA on skeletal muscle atrophy in the skeletal muscle of aged HFD-fed mice. (**A**) Western blots of the atrophic proteins p-FoxO3a, FoxO3a, Atrogin-1, MuRF1, and Myostatin in the QUA. (**B**) Western blots of atrophic proteins in the GAS. Values with different letters are significantly different: *p* < 0.05 (a > b > c > d > e > f).

Both the PI3K/Akt pathway and ubiquitin-mediated protein degradation are essential for the maintenance of skeletal muscle [35]. FoxO3a phosphorylation inhibits the expression of its targets, Atrogin-1 and MuRF1, which are the key proteins of the ubiquitin-proteasome system. The phosphorylation of FoxO3a was decreased in the QUA and GAS of aged HFD-fed mice, and accordingly, protein levels of Atrogin-1, MuRF1, and myostatin were increased in aged HFD-fed mice compared to young HFD-fed mice and aged CD-fed mice (Figure 8A,B). However, GABA treatment significantly increased p-FoxO3a levels and decreased the expression of Atrogin-1, MuRF1, and myostatin in both the QUA and GAS. Together, these findings indicated that GABA decreased sarcopenia by suppressing muscle protein degradation in aged HFD-fed mice.

### 3.9. GABA Increased Muscle Fiber Size and Myogenesis in Aged Mice

**Figure 9:**
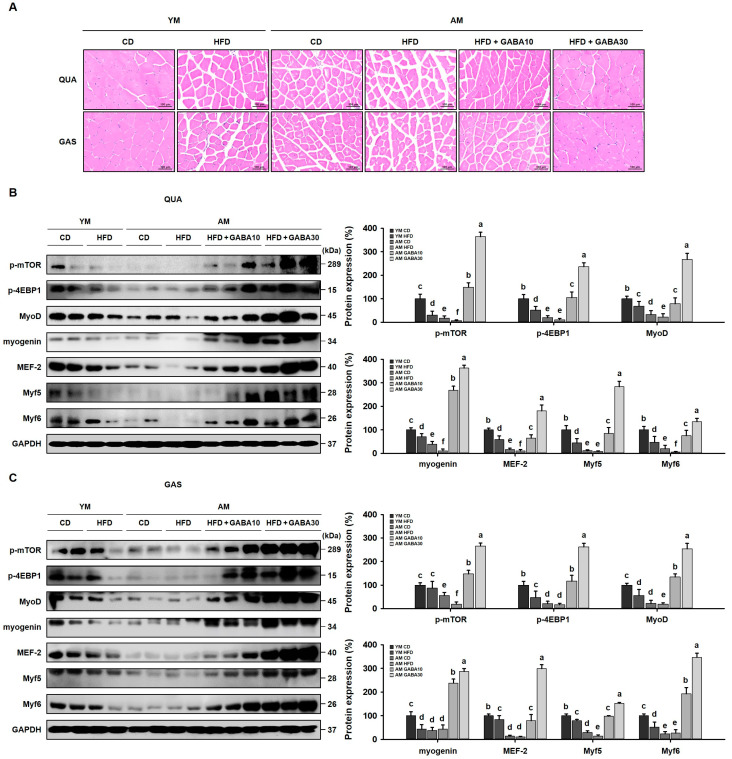
Effects of GABA on myogenesis in the skeletal muscle of aged HFD-fed mice. (**A**) H&E staining of the QUA and GAS. Scale bar: 100 μm. (**B**) Western blots of QUA myogenic proteins, including p-mTOR, p-4EBP1, MyoD, myogenin, MEF-2, Myf5, and Myf6. (**C**) Western blots of GAS myogenic protein levels. Values with different letters are significantly different: *p* < 0.05 (a > b > c > d > e > f).

**Figure 10:**
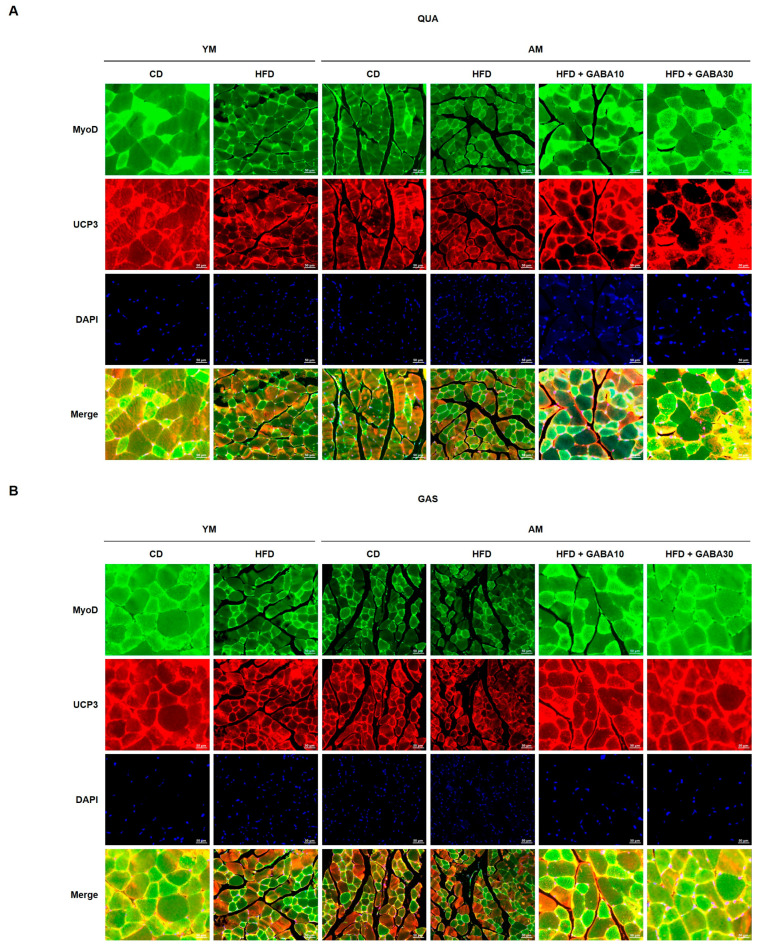
Double immunofluorescence staining of skeletal muscle for myogenesis and energy expenditure. (**A**) Immunofluorescence images of the expression of MyoD (green) and UCP3 (red) in the QUA and (**B**) GAS. Scale bar: 50 μm.

Myogenesis, the synthesis and regeneration of skeletal muscle fiber, is important for the restoration of skeletal muscle mass and functions following muscle atrophy [36]. Histological analyses of the QUA and GAS revealed that the HFD and aging decreased the muscle fiber size (Figure 9A). In GABA-treated mice, the cross-sectional muscle fiber area was significantly increased. Consistent with these findings, mTOR-4EBP1 interactions and myogenic regulatory factors (MRFs) are activators of myogenesis, so we subsequently determined whether GABA treatment increased the phosphorylation of mTOR-4EBP1 and MRFs, including MyoD, myogenin, MEF-2, Myf5, and Myf6. GABA treatment increased the expression of myogenic proteins in GABA-treated aged HFD-fed mice (Figure 9B,C). Furthermore, GABA increased QUA and GAS MyoD and UCP3 levels in aged HFD-fed mice, as demonstrated via immunofluorescence (Figure 10A,B). Taken together, these findings suggest that GABA increased the muscle fiber size and myogenesis by inducing muscle protein synthesis in aged mice.

### 3.10. GABA Decreases Pro-Inflammatory Cytokines and Increases Anabolic Hormones in Aged Mice

**Figure 11:**
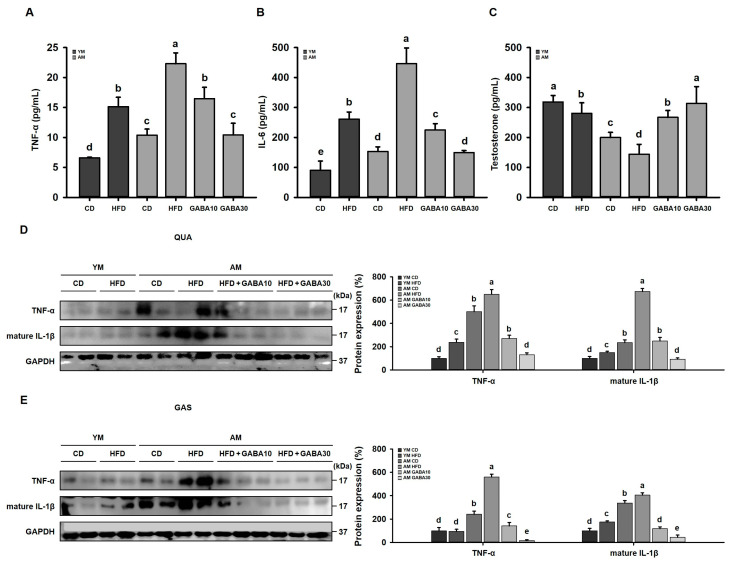
Effects of GABA on pro-inflammatory cytokines and anabolic hormones in aged HFD-fed mice. (**A**) Pro-inflammatory cytokine TNF-α and (**B**) IL-6 concentrations after 8 weeks. (**C**) Testosterone concentration. (**D**) Western blots of the pro-inflammatory mediators TNF-α and mature IL-1β in the QUA. (**E**) Western blots of inflammatory mediators in the GAS. Values with different letters are significantly different: *p* < 0.05 (a > b > c > d > e).

Obesity- and age-related disturbance of immune homeostasis cause chronic low-grade inflammation, a primary cause of sarcopenic obesity [37]. Therefore, we investigated the effect of GABA on inflammatory markers in both the serum and skeletal muscle. The pro-inflammatory cytokines TNF-α and IL-6 were significantly higher in the serum of aged HFD-fed mice than in young HFD-fed mice and aged CD-fed mice (Figure 11A,B), indicating that sarcopenic obesity promotes chronic low-grade inflammation. GABA treatment significantly decreased serum levels of pro-inflammatory cytokines in aged HFD-fed mice. In sarcopenic obesity, inflammation is closely linked to the anabolic hormone testosterone. Pro-inflammatory cytokines were increased in aged HFD-fed mice, while testosterone was decreased (Figure 11C). However, GABA treatment increased serum testosterone levels. Moreover, in the QUA and GAS, protein levels of TNF-α and mature IL-1β in aged HFD-fed mice were higher than in young mice but decreased in GABA-treated groups (Figure 11D,E). These findings indicate that GABA treatment decreases HFD- and age-related chronic inflammation and increases testosterone, which maintains skeletal muscle function and muscle protein turnover in aged mice.

## 4. Discussion

Obesity and aging are associated with sarcopenia, which is regulated by complex processes, including insulin resistance, mitochondrial dysfunction, and inflammation, which cause fat mass gain and muscle mass loss [38]. Sarcopenic obesity, the co-occurrence of both obesity and sarcopenia, is becoming a major public health burden as the population ages [39]. Increased awareness of the importance of healthy aging has prompted investigations of dietary and pharmacological interventions to prevent or alleviate sarcopenic obesity. In the present study, we newly identified the therapeutic efficacy and regulatory mechanisms of GABA in the context of sarcopenic obesity. The first objective of the study was to determine differences in body composition between young and aged mice and how the interrelated pathologies of obesity and aging interact to precipitate the loss of skeletal muscle mass and resultant sarcopenia. Therefore, we aimed to examine the potential links between adipose tissue and skeletal muscle, the two primary tissues affected by age- and obesity-associated changes in body composition.

We developed a mouse model of sarcopenic obesity by combining aging-associated sarcopenia and HFD-induced obesity. HFD feeding induces obesity and increases body mass and fat mass. These changes in body fat mass resulted in changes in the general metabolic status. For fat mass, we measured both visceral and subcutaneous fat. DEXA and H&E staining analysis identified that the HFD increased adipogenesis in both young and aged mice, especially in the VAT, which is metabolically active. However, the effect of the HFD on aged mice was much more robust, suggesting that aging exacerbates obesity. Importantly, visceral obesity increases the risk and severity of metabolic syndrome, including insulin resistance and dyslipidemia, more significantly than subcutaneous obesity in the elderly [40]. Therefore, to further examine the effect and mechanism of GABA on lipid metabolism and insulin resistance, VAT was analyzed.

HFD significantly increases VAT accumulation, which was concomitant with increased adipogenic proteins (C/EBPα, PPARγ, and FABP4) and lipogenic proteins (SREBP1, LPAATθ, lipin1, and DGAT1). However, body mass gain and VAT lipid accumulation were decreased by GABA treatment in aged HFD-fed mice. Moreover, other important physiological changes, such as a body temperature decrease, occur with age [41]. Consistently, we identified in the present study that body temperature was decreased in aged mice relative to that in young mice. Further, aged HFD-fed mice lost their thermogenic capacity. However, GABA significantly increased the body temperature in aged HFD-fed mice, suggesting the animals generated more energy as heat. Consistent with this finding, we identified that GABA stimulated energy expenditure by releasing free fatty acids via lipolysis, which are then oxidized in the mitochondria, finally promoting VAT browning. In support of this finding, in GABA-treated aged HFD-fed mice, free fatty acids released during lipolysis by lipolytic proteins, including p-PKA, ATGL, p-HSL, and MGL, were transported into the mitochondria, which dissipates energy as heat via the UCP1-mediated browning pathway. In addition, we demonstrated that GABA induces PKA and thus activates UCP1 immunoreactivities in the VAT of aged HFD-fed mice, suggesting that GABA enhances energy expenditure through lipolysis and thermogenesis.

The interrelationship between obesity in adipose tissue and the atrophy of skeletal muscle drives the pathology of sarcopenic obesity [42,43]. Further, skeletal muscle is energetically demanding, requiring significant quantities of ATP for the contractility of muscle fibers. ATP is produced primarily by mitochondria in skeletal muscle [44]. Mitochondrial dysfunction results in imbalances in energy metabolism, a major contributor to sarcopenic obesity [45]. Skeletal muscle mitochondrial function was decreased by HFD feeding, which is consistent with the onset of sarcopenic obesity. Moreover, our findings demonstrated that HFD-induced skeletal muscle mitochondrial dysfunction was more severe in aged mice. GABA treatment increased the expression of mitochondrial biogenesis markers, such as PGC1α, NRF1, TFAM, and a mitochondrial inner membrane transporter, UCP3, in the QUA and GAS of aged HFD-fed mice. This suggests that GABA promotes mitochondrial activity and contributes to energy production not only in adipose tissue but also in skeletal muscle.

Adipose tissue and skeletal muscle regulate lipid and glucose metabolism. HFD-induced obesity contributes to hyperlipidemia and hyperglycemia in both adipose tissue and skeletal muscle, which contributes to muscle atrophy [46]. GABA alleviated obesity-induced increases in fasting blood glucose, improved glucose tolerance and insulin resistance, and improved the serum lipid profile, including decreased TG, TC, and LDL and increased HDL, suggesting that GABA improves insulin sensitivity. Insulin resistance disturbs regulation of the PI3K/Akt pathway, which is a classic glucose metabolism pathway in many organs, including adipose tissue and skeletal muscle [47]. Our findings revealed that GABA promotes glucose uptake through the insulin-mediated activation of the PI3K/Akt pathway and subsequent cellular membrane translocation of GLUT4. GABA activated this mechanism in both the VAT and skeletal muscle of aged HFD-fed mice, suggesting that GABA supports glucose homeostasis and insulin sensitivity.

The insulin–PI3K/Akt pathway also suppresses muscle degradation by phosphorylating and inactivating FoxO3a, which transcribes the muscle wasting factors Atrogin-1 and MuRF1 [48]. Interestingly, we observed similar patterns of DEXA and H&E staining in young obese mice and aged non-obese mice, revealing that both obesity and aging decrease the skeletal muscle mass and fiber size. Moreover, we demonstrated a greater loss of muscle mass and fiber size in aged obese (sarcopenic obesity) mice than in animals challenged with either obesity or aging alone. Similarly, FoxO3a inhibits muscle protein degradation, and GABA treatment decreased protein levels of muscle atrophy-related ubiquitin ligases, including Atrogin-1 and MuRF1, in aged HFD-fed mice. These findings demonstrated that not only aging, but also obesity, could induce skeletal muscle atrophy. Furthermore, a sarcopenic obese state is more severe than obesity or sarcopenia alone. GABA inhibited obesity- and age-related muscle protein degradation via activation of the PI3K/Akt pathway, which suppresses the ubiquitin-proteasome pathway.

The balance between muscle protein degradation and synthesis is crucial for maintaining skeletal muscle mass and function [49]. The myogenic differentiation process interacts with skeletal muscle fibers and is essential for muscle regeneration following muscle atrophy. Therefore, we also investigated the muscle protein synthesis pathway related to mTOR-mediated myogenic signaling regulatory factors to evaluate the effect of GABA on myogenic activation. GABA increased phosphorylation of the mTOR-4EBP1 interactions and protein levels of myogenic differentiation transcription factors, including MyoD, myogenin, MEF-2, Myf5, and Myf6, in the skeletal muscle of HFD-fed aged mice, suggesting that GABA alleviates obesity- and age-induced sarcopenia by promoting muscle regeneration.

Obesity induces a chronic low-grade inflammatory state, as does inflammaging, defined as age-related increases in pro-inflammatory cytokines, including TNF-α, IL-1β, and IL-6 [50]. Both processes are significant risk factors for sarcopenic obesity. Consistently, in the present study, we identified that both serum and tissue protein levels of pro-inflammatory cytokines were higher in obese aged mice than in mice with obesity or sarcopenia alone. GABA effectively decreased inflammation due to both obesity and sarcopenia. Moreover, sarcopenic obesity causes pathological hormonal changes, including decreases in the anabolic hormone testosterone. Indeed, we observed decreases in testosterone in aged HFD-fed mice, which likely impeded muscle growth. In GABA-treated aged HFD-fed mice, serum levels of testosterone were significantly increased relative to those in untreated aged HFD-fed mice. Taken together, we postulate that obesity and aging synergistically induce sarcopenic obesity by increasing pro-inflammatory cytokines and decreasing anabolic hormones.

## 5. Conclusions

In conclusion, these findings demonstrated that obesity and aging contribute synergistically to skeletal muscle atrophy in the process of sarcopenic obesity, which significantly decreases quality of life in the elderly. This is an important molecular mechanism underlying the progression of both obesity and sarcopenia simultaneously in aged mice. Although the anti-sarcopenic effect of GABA in aged mice or anti-obesity effect of GABA in young mice has not yet been reported, the present study identified GABA as a novel approach for sarcopenic obesity using GABA in aged HFD-fed obese mice. We revealed that GABA could potentially be developed as an anti-sarcopenic obesity agent, as GABA suppresses fat accumulation and muscle degradation and increases energy expenditure and muscle protein synthesis. Therefore, it is expected that GABA could be used as an effective therapeutic intervention for sarcopenic obesity and potentially for obesity- and aging-related metabolic syndrome.

## Data Availability

All data generated or analysed during this study are included in this published article.
